# Supplementary material for: Emergence and Spread of B.1.1.7 Lineage in Primary Care and Clinical Impact in the Morbi-Mortality among Hospitalized Patients in Madrid, Spain
Source: Microorganisms. 2021 Jul 15;9(7):1517. doi: 10.3390/microorganisms9071517 (PMC8307589; doi:10.3390/microorganisms9071517)
Supplement: Supplementary file 1 [file microorganisms-09-01517-s001.zip › Table S1.pdf]

Table S1. Accession numbers available in GISAID of SARS-CoV-2 sequences belonging to B.1.1.7 lineage from COVID-19 patients detected during the period of study.

Screening	Sequencing	GISAID access number	Date of sampling
UK variant	B.1.1.7	EPI_ISL_882617	Dec 2020
UK variant	B.1.1.7	EPI_ISL_882621	Dec 2020
UK variant	B.1.1.7	EPI_ISL_882623	Dec 2020
UK variant	B.1.1.7	EPI_ISL_882612	Jan 2021
UK variant	B.1.1.7	EPI_ISL_882613	Jan 2021
UK variant	B.1.1.7	EPI_ISL_882615	Jan 2021
UK variant	B.1.1.7	EPI_ISL_882616	Jan 2021
UK variant	B.1.1.7	EPI_ISL_882618	Jan 2021
UK variant	B.1.1.7	EPI_ISL_882620	Jan 2021
UK variant	B.1.1.7	EPI_ISL_882624	Jan 2021
UK variant	B.1.1.7	EPI_ISL_882625	Jan 2021
UK variant	B.1.1.7	EPI_ISL_882627	Jan 2021
UK variant	B.1.1.7	EPI_ISL_882628	Jan 2021
UK variant	B.1.1.7	EPI_ISL_882629	Jan 2021
UK variant	B.1.1.7	EPI_ISL_882631	Jan 2021
UK variant	B.1.1.7	EPI_ISL_882632	Jan 2021
UK variant	B.1.1.7	EPI_ISL_931584	Feb 2021
UK variant	B.1.1.7	EPI_ISL_933718	Feb 2021
UK variant	B.1.1.7	EPI_ISL_933721	Feb 2021
UK variant	B.1.1.7	EPI_ISL_933723	Feb 2021
UK variant	B.1.1.7	EPI_ISL_933725	Feb 2021
UK variant	B.1.1.7	EPI_ISL_933727	Feb 2021
UK variant	B.1.1.7	EPI_ISL_933728	Feb 2021
UK variant	B.1.1.7	EPI_ISL_933729	Feb 2021
UK variant	B.1.1.7	EPI_ISL_933730	Feb 2021
UK variant	B.1.1.7	EPI_ISL_933731	Feb 2021
UK variant	B.1.1.7	EPI_ISL_933733	Feb 2021
UK variant	B.1.1.7	EPI_ISL_933734	Feb 2021
UK variant	B.1.1.7	EPI_ISL_933740	Feb 2021
UK variant	B.1.1.7	EPI_ISL_933767	Feb 2021
UK variant	B.1.1.7	EPI_ISL_933768	Feb 2021
UK variant	B.1.1.7	EPI_ISL_933770	Feb 2021
UK variant	B.1.1.7	EPI_ISL_933771	Feb 2021
UK variant	B.1.1.7	EPI_ISL_933773	Feb 2021
UK variant	B.1.1.7	EPI_ISL_933774	Feb 2021
UK variant	B.1.1.7	EPI_ISL_1665128	Mar 2021
UK variant	B.1.1.7	EPI_ISL_1665130	Mar 2021
UK variant	B.1.1.7	EPI_ISL_1665132	Mar 2021
UK variant	B.1.1.7	EPI_ISL_1665133	Mar 2021
UK variant	B.1.1.7	EPI_ISL_1665134	Mar 2021
UK variant	B.1.1.7	EPI_ISL_1665137	Mar 2021

UK variant	B.1.1.7	EPI_ISL_1665140	Mar 2021
UK variant	B.1.1.7	EPI_ISL_1665141	Mar 2021
UK variant	B.1.1.7	EPI_ISL_1665142	Mar 2021
UK variant	B.1.1.7	EPI_ISL_1665143	Mar 2021
UK variant	B.1.1.7	EPI_ISL_1665193	Mar 2021
UK variant	B.1.1.7	EPI_ISL_1665196	Mar 2021
UK variant	B.1.1.7	EPI_ISL_1665197	Mar 2021
UK variant	B.1.1.7	EPI_ISL_1665238	Mar 2021
UK variant	B.1.1.7	EPI_ISL_1665239	Mar 2021
UK variant	B.1.1.7	EPI_ISL_1665240	Mar 2021
UK variant	B.1.1.7	EPI_ISL_1665241	Mar 2021
UK variant	B.1.1.7	EPI_ISL_1665242	Mar 2021
UK variant	B.1.1.7	EPI_ISL_1665244	Mar 2021
UK variant	B.1.1.7	EPI_ISL_1665253	Mar 2021
UK variant	B.1.1.7	EPI_ISL_1665270	Mar 2021
UK variant	B.1.1.7	EPI_ISL_1665271	Mar 2021
UK variant	B.1.1.7	EPI_ISL_1665275	Mar 2021
UK variant	B.1.1.7	EPI_ISL_1665276	Mar 2021
UK variant	B.1.1.7	EPI_ISL_1665277	Mar 2021
UK variant	B.1.1.7	EPI_ISL_1665279	Mar 2021
UK variant	B.1.1.7	EPI_ISL_1665280	Mar 2021
UK variant	B.1.1.7	EPI_ISL_1912208	Apr 2021
UK variant	B.1.1.7	EPI_ISL_1912210	Apr 2021
UK variant	B.1.1.7	EPI_ISL_1912213	Apr 2021
UK variant	B.1.1.7	EPI_ISL_1912216	Apr 2021
UK variant	B.1.1.7	EPI_ISL_1912219	Apr 2021
UK variant	B.1.1.7	EPI_ISL_1912222	Apr 2021
UK variant	B.1.1.7	EPI_ISL_1912224	Apr 2021
UK variant	B.1.1.7	EPI_ISL_1912227	Apr 2021
UK variant	B.1.1.7	EPI_ISL_1912233	Apr 2021
UK variant	B.1.1.7	EPI_ISL_1912236	Apr 2021
UK variant	B.1.1.7	EPI_ISL_1912239	Apr 2021
UK variant	B.1.1.7	EPI_ISL_1912244	Apr 2021
UK variant	B.1.1.7	EPI_ISL_1912247	Apr 2021
UK variant	B.1.1.7	EPI_ISL_1912250	Apr 2021
UK variant	B.1.1.7	EPI_ISL_1912252	Apr 2021
UK variant	B.1.1.7	EPI_ISL_1912255	Apr 2021
UK variant	B.1.1.7	EPI_ISL_1912258	Apr 2021
UK variant	B.1.1.7	EPI_ISL_1912260	Apr 2021
UK variant	B.1.1.7	EPI_ISL_1912263	Apr 2021
UK variant	B.1.1.7	EPI_ISL_1912266	Apr 2021
UK variant	B.1.1.7	EPI_ISL_1912269	Apr 2021
UK variant	B.1.1.7	EPI_ISL_1912271	Apr 2021
UK variant	B.1.1.7	EPI_ISL_1912277	Apr 2021
UK variant	B.1.1.7	EPI_ISL_1912282	Apr 2021

UK variant	B.1.1.7	EPI_ISL_1912285	Apr 2021
UK variant	B.1.1.7	EPI_ISL_1912287	Apr 2021
UK variant	B.1.1.7	EPI_ISL_1912290	Apr 2021
UK variant	B.1.1.7	EPI_ISL_1912293	Apr 2021
UK variant	B.1.1.7	EPI_ISL_1912299	Apr 2021
UK variant	B.1.1.7	EPI_ISL_1912302	Apr 2021
UK variant	B.1.1.7	EPI_ISL_1912308	Apr 2021
UK variant	B.1.1.7	EPI_ISL_1912310	Apr 2021
UK variant	B.1.1.7	EPI_ISL_1912315	Apr 2021
UK variant	B.1.1.7	EPI_ISL_2104411	Apr 2021
UK variant	B.1.1.7	EPI_ISL_2104412	Apr 2021
UK variant	B.1.1.7	EPI_ISL_2104413	Apr 2021
UK variant	B.1.1.7	EPI_ISL_2104414	Apr 2021
UK variant	B.1.1.7	EPI_ISL_2104416	Apr 2021
