# Supplementary material for: Emergence and Spread of B.1.1.7 Lineage in Primary Care and Clinical Impact in the Morbi-Mortality among Hospitalized Patients in Madrid, Spain
Source: Microorganisms. 2021 Jul 15;9(7):1517. doi: 10.3390/microorganisms9071517 (PMC8307589; doi:10.3390/microorganisms9071517)
Supplement: Supplementary file 1 [file microorganisms-09-01517-s001.zip › Table S2.pdf]

Table S2. Demographic and clinical characteristics of the patients infected by B.1.1.7 and non-B.1.1.7 lineages according to age group (≤65 and >65 year). IQR: interquartile range, OR: odds ratio, 95%CI: 95% confidence interval, ICU: intensive care unit, IRCU: intensive respiratory care unit, sig: significance.

Variable	Patients ≤65 Years				Patients >65 Years			
	**B.1.1.7/SGTF (*n* = 191)**	**B.1.1.7 (*n* = 453)**	***p***	**OR**	**B.1.1.7/SGTF (*n* = 235)**	**B.1.1.7 (*n* = 676)**	***p***	**OR**
**Age (median)**	54	54	0.62		78	82	**0.001**	
**IQR**	(45–60)	(47–60)			(71–86)	(74–88)		
**Sex**								
**Women**	36.1%	36.9%	0.86		45.1%	49.3%	0.27	
**Men**	63.9%	63.1%			54.9%	50.7%		
**ICU admission**	23.6%	18.1%	0.11	1.39	16.2%	5.0%	**0.001**	3.64
**(95%CI)**	(17.73–30.23)	(14.66–21.96)		(0.92–2.1)	(11.7–21.51)	(3.5–6.96)		(2.23–5.94)
**IRCU admission**	4.2%	1.8%	0.07	2.43	12.3%	6.2%	**0.03**	2.12
**(95%CI)**	(1.82–8.09)	(0.76–3.45)		(0.89–6.58)	(8.42–17.24)	(4.51–8.3)		(1.29–3.49)
**Death**	4.2%	3.5%	0.68	1.21	22.5%	23.6%	0.57	0.82
**(95%CI)**	(1.69–8.39)	(1.96–5.66)		(0.48–3.02)	(16.79–29.1)	(20.37–27.03)		(0.64–1.38)
